# Variation Trends of Fine Particulate Matter Concentration in Wuhan City from 2013 to 2017

**DOI:** 10.3390/ijerph15071487

**Published:** 2018-07-13

**Authors:** Daoru Liu, Qinli Deng, Zeng Zhou, Yaolin Lin, Junwei Tao

**Affiliations:** 1 School of Civil Engineering and Architecture, Wuhan University of Technology, Wuhan 430070, China; 228640@whut.edu.cn; 2 School of Urban Design, Wuhan University, Wuhan 430070, China; 3 School of Environmental Science and Technology, Huazhong University of Science and Technology, Wuhan 430070, China; m201773713@hust.edu.cn

**Keywords:** natural day, distribution, time span, pattern, primary pollutant

## Abstract

Fine particulate matter (PM_2.5_) is directly associated with smog and has become the primary factor that threatens air quality in China. In order to investigate the variation patterns of PM_2.5_ concentrations in various regions of Wuhan city across different time spans, we analyzed continuous monitoring data from six monitoring sites in Wuhan city from 2013 to 2017. The results showed that the PM_2.5_ concentration from the various monitoring sites in the five-year period showed a decreasing trend. January, October, and December are the three months with relatively high mean monthly PM_2.5_ concentrations in the year, while June, July, and August are the three months with relatively low mean monthly PM_2.5_ concentrations in the year. The number of days with a daily mean concentration of 35–75 μg/m^3^ was the highest, while the number of days with a daily mean concentration of more than 250 μg/m^3^ was the lowest. PM_2.5_ accounted for a large proportion of the major pollutants and is the main source of air pollution in Wuhan city, with an average proportion of over 46%.

## 1. Introduction

With rapid industrialization and urbanization, the problem of air pollution, particularly the problem of smog, has become increasing severe. PM_2.5_ is the particulate matter with an aerodynamic diameter less than or equal to 2.5 μm, which is directly associated with smog and has become the primary factor that threatens air quality in China [1]. PM_2.5_ pollution in China has a relatively clear spatial and temporal distribution, with severe pollution mainly occurring during the transition from autumn to winter and winter to spring [2]. PM_2.5_ contains several harmful substances, and the particles of PM_2.5_ enter the lungs through the respiratory system and threaten human health, particularly that of children and the elderly. In recent years, China has experienced multiple outbreaks of respiratory diseases, which have resulted in school closures and factory shutdowns, thus causing social panic and economic losses. At the same time, the problem of smog is showing a trend of spreading from north regions to south regions in China. The air quality of Wuhan has been deteriorating, and PM_2.5_ has become the city’s primary air pollutant [2].

In recent years, Chinese researchers have conducted PM_2.5_ studies that have been focused on the spatial distribution, composition, source, and transmission laws of PM_2.5_ [3,4,5,6,7,8]. Jiao et al. employed mathematical statistics and geographic information system (GIS) spatial analysis to analyze the temporal variation and spatial distribution characteristics of PM_2.5_. Their results showed that spatial heterogeneity of the PM_2.5_ concentration is greater in summer than in winter, and the pollution is severe in industrial regions and densely populated regions than in green spaces and parks in the city. The consistency in the variation in the degree of pollution is associated with distance to industrial regions and the surrounding environment [9]. Cho et al. reviewed in vitro and in vivo experimental studies of PM_2.5_ in the progression of various diseases from the last decade. Oxidative stress, inflammation, and genotoxicity are considered as the main potential mechanisms in PM_2.5_-induced disease progression in their conclusion [10]. Ho et al. adopted positive matrix factorization (PMF) to explore and examine the proportion of each source that contributed to the total PM_2.5_ concentration. Their conclusion raveled the relationship between pollutants released by pollution sources and secondary pollution [11]. Zhang et al. employed inductively coupled plasma mass spectrometry for studying the characteristics of trace elements and rare earth elements in PM_2.5_ in Wuhan city. The results showed that the majority of the pollution sources of PM_2.5_ in Wuhan city were artificial pollution sources, and the majority of the rare earth elements in PM_2.5_ were from soil pollution sources [12,13]. Zhang et al. proposed a regression model using the Eigenvector Spatial Filtering method to estimate ground PM_2.5_ concentration. The results proved that the ESF-based Regression model is an effective approach to analyze and predict the PM_2.5_ concentration [14]. Cao et al. used the actual measurement data of water-soluble ions in atmospheric PM_2.5_ and automatically monitored the fine-particle data of Wuhan city in order to analyze the atmospheric pollution characteristics in Wuhan during the severe pollution observed in autumn and winter and during cleaning. The results showed that PM_2.5_ is the primary atmospheric pollutant during autumn and winter in Wuhan [15]. Ma and Cheng et al. conducted studies on PM_2.5_ carbon characteristics in different temporal and spatial ranges in Wuhan city [16,17]. Yang et al. used a log-linear exposure–response model and monetary valuation methods, such as value of a statistical life, amended human capital, and cost of illness, to evaluate PM_2.5_-related economic losses from health impacts at the city level. In addition, a Monte Carlo simulation was used to analyze uncertainty, the results of which showing that the increase in PM_2.5_ concentration poses a serious threat to urban economic development and residents’ health [18]. Ma et al. made use of the hourly PM_2.5_ mass concentration from ground-based observations in Beijing-Tianjin-Hebei. The results showed long-term monitoring data can be utilized to precisely predict future PM_2.5_ concentration [19].

Foreign researchers have also conducted large-area and multi-scale PM_2.5_ studies [20,21,22,23,24,25,26]. Aboubacar et al. investigated the impact of PM_2.5_ from household combustion on life expectancy considering several covariates while controlling for ambient PM_2.5_ generated by other sectors. In addition, the generalized method of moments model and the panel cointegration model were applied to a dataset of 43 Sub-Saharan Africa countries over the time period of 1995–2010. The results indicated household PM_2.5_ is significantly and negatively associated with higher aggregate life expectancy in the long-run, and, to a greater degree for female’s [27]. Phung et al. utilized daily emergency ambulance dispatches data from eight Japanese cities (2007–2011) and conducted two-dimensional statistical analyses. The results demonstrated PM_2.5_ has a considerable effect on all-cause, respiratory, and neuropsychological emergency ambulance dispatches [28]. Jugder and Shinoda et al. conducted a 16-month monitoring study of four monitoring sites in Mongolia. The results showed that sandstorms during spring and winter are the root cause of PM_2.5_ and PM_10_ (particulate matter with an aerodynamic diameter less than or equal to 10 μm) elevations in that region. During sandstorms, PM_2.5_ concentrations in the atmosphere increase greatly [29]. Baker and Foley proposed a non-linear regression model based on photochemical transport models. Their model could effectively estimate the primary and secondary PM_2.5_ concentrations from a single source [30]. Based on diagnostic ratios of carbonaceous species, Genga et al. researched the mineral dust, organic carbon, elemental carbon, water-soluble organic carbon, sea salts, and anthropogenic metals in Brindisi, and assessed the presence of biomass burning emissions, fossil fuel emissions, and ship emission. The results showed that the existence of aged combustion aerosols can influence the measured data to a considerable extent [31]. Ju conducted a study from an international trade perspective and employed a structural path analysis for the quantization of transboundary PM_2.5_ and the tracking of transaction paths, identified the relationship between the consumption and production responsibilities of China, Japan, and Korea, and reviewed existing environmental cooperation mechanisms and policies. The results showed that Japan and South Korea are strongly dependent on China economically and various countries should actively participate in the governance of environmental problems such that all the participating countries benefit [32]. Aldabe et al. collected the data of PM_2.5_ and PM_10_ from three different areas (rural, urban, and urban-traffic) in Navarra, Spain. They performed PMF model to identify five principle sources for PM_10_ and PM_2.5_ in Iturrama and Plaza de la Cruz, and 4 sources for PM_10_ in Bertiz [33]. Siskos et al. employed a Harvard impactor system to simultaneously collect atmospheric aerosols and PM_2.5_ data in Greece. Ion chromatography and a semi-micro electrodes were used to measure the chemical composition and aerosol acidity in samples, respectively. The analysis results showed that the majority of aerosol ions near the sea were acidic, but that their overall concentration was low. Aerosol ions in city centers were neutral but their overall concentrations were high. At the same time, ion concentrations were higher in spring and winter throughout the year [34]. Saliba et al. conducted a long-term assessment of PM_2.5_ and PM_10_ concentration in Eastern Mediterranean region. The results showed that the semi-enclosed structure and vehicle emissions lead to higher PM_2.5_ and PM_10_ concentrations in the region [35]. Singh et al. employed an Andersen cascade impactor sampler to collect atmospheric organic aerosols from Delhi during January 2006 to December 2007 in order to study the particle distribution in aerosols and their seasonal characteristics. The obtained results showed that the burning of biofuels and biomass as well as other human activities significantly increase PM_2.5_ concentrations. Autumn and winter are the two seasons in which PM_2.5_ concentrations are higher in the year. The over-limit status of PM_2.5_ concentrations throughout the year is an issue [36]. Rao et al. performed a bootstrap simulation analysis to estimate the uncertainty of the emission of wood consumption and emission factors of different appliance types [37]. Pipal et al. collected PM_2.5_ and PM_10_ samples from various monitoring sites in Agra in order to study the yearly variation trends of mineral aerosols. The results of the study showed that there are diverse sources of local PM_2.5_, of which crustal activity and local traffic and transportation are the primary sources [38]. Foreign studies on PM_2.5_ tend to focus on composition, source, and economics, and there are few studies on the long-term variation patterns of PM_2.5_.

PM_2.5_ governance has become a social consensus, but the prerequisite for governance requires an understanding of PM_2.5_, and its causes, composition, and temporal and spatial distribution patterns require research. The majority of PM_2.5_ studies in Wuhan have a one-year timespan, and there is a lack of studies on long-term concentration variation trends, which is necessary for PM_2.5_ governance. In this study, we analyzed the PM_2.5_ data from various regions in Wuhan city in the period of 2013–2017 and its concentration variation trends for different timescales. This will provide a reference for PM_2.5_ governance for different periods in various regions in Wuhan city.

## 2. Sample Collection, Analysis and Calculation

**Figure 1:**
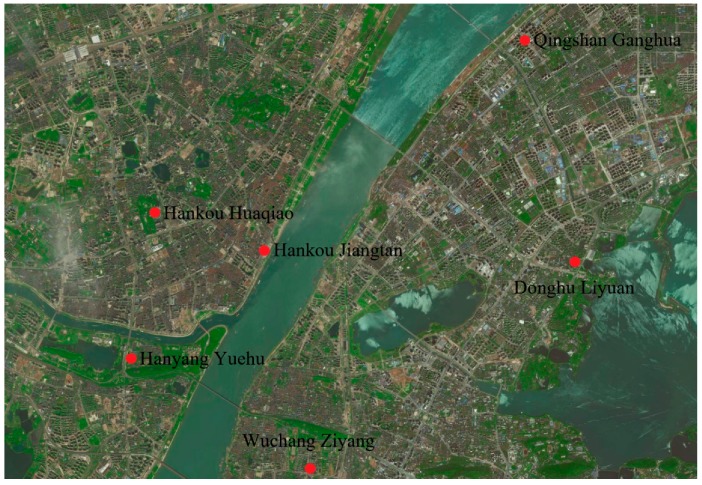
Distribution of monitoring sites.

Currently, Wuhan City has a total of 32 automatic ambient air quality monitoring sites, including 10 state-controlled monitoring sites, 11 city-controlled monitoring sites, 4 region-controlled monitoring sites, 1 combined atmospheric pollution automatic monitoring laboratory, 1 roadside site, 1 control site, and 4 regional sites. The state-controlled monitoring sites (Hanyang Yuehu, Hankou Huaqiao, Hankou Jiangtan, Wuchang Ziyang, Donghu Liyuan, and Qingshan Ganghua) were used for the analysis. The data in this study was obtained from the Wuhan Environmental Protection Bureau. Figure 1 shows the distribution of the monitoring sites. Sample collection and analysis were performed according to standards [39]. Table 1 shows the device range and precision information. Glass fiber filters were used because their retention efficiency with 0.3 μm-standard particles is higher than 99%. The sensitivity of the analytical balance was 0.01 mg. The thermostatic incubator temperature was set to 15–30 °C, and the temperature precision was ±1 °C. The relative humidity was adjusted to 50 ± 5%.

The sampler entrance is 1.5 m above the ground, and the sampling points are kept away from direct pollution sources and obstacles. An intermittent sampling at intervals of 5 h was used to measure the daily mean concentration. After sampling, the filters were weighed according to standards. Samples that could not be weighed immediately were labeled and stored at 4 °C. The PM_2.5_ concentration was calculated using Equation (1). During the data processing, if more than two data points were missing in a day, the day’s data was considered to be invalid. If data from more than 6 days were missing in a month, that month’s data was considered to be invalid. If the data for more than 3 months were missing, that year’s data was considered to be invalid [2,16,40].

(1) $$\;\rho =\frac{w_{2}-w_{1}}{V}\;$$

In the equation, *ρ* is the PM_2.5_ concentration in mg/m^3^ (converted to μg/m^3^ for statistical analysis);

*w*_1_ = weight of blank filter in g;

*w*_2_ = weight of filter after sampling in g;

*V* = sample volume after conversion to standard conditions (101.325 kPa and 273 K) in m^3^.

## 3. Results and Discussion

### 3.1. Annual Variation Trend of PM_2.5_ Concentrations

Table 2 shows the valid hours and validity rate for PM_2.5_ concentration monitoring data for the period of 2013–2017. As seen from the table, the minimum value for the validity rate for the monitoring data at all the sites in the 5-year period was 93.4%, and the maximum value was 99.7%. The data reliability was high and could be used in this paper.

**Figure 2:**
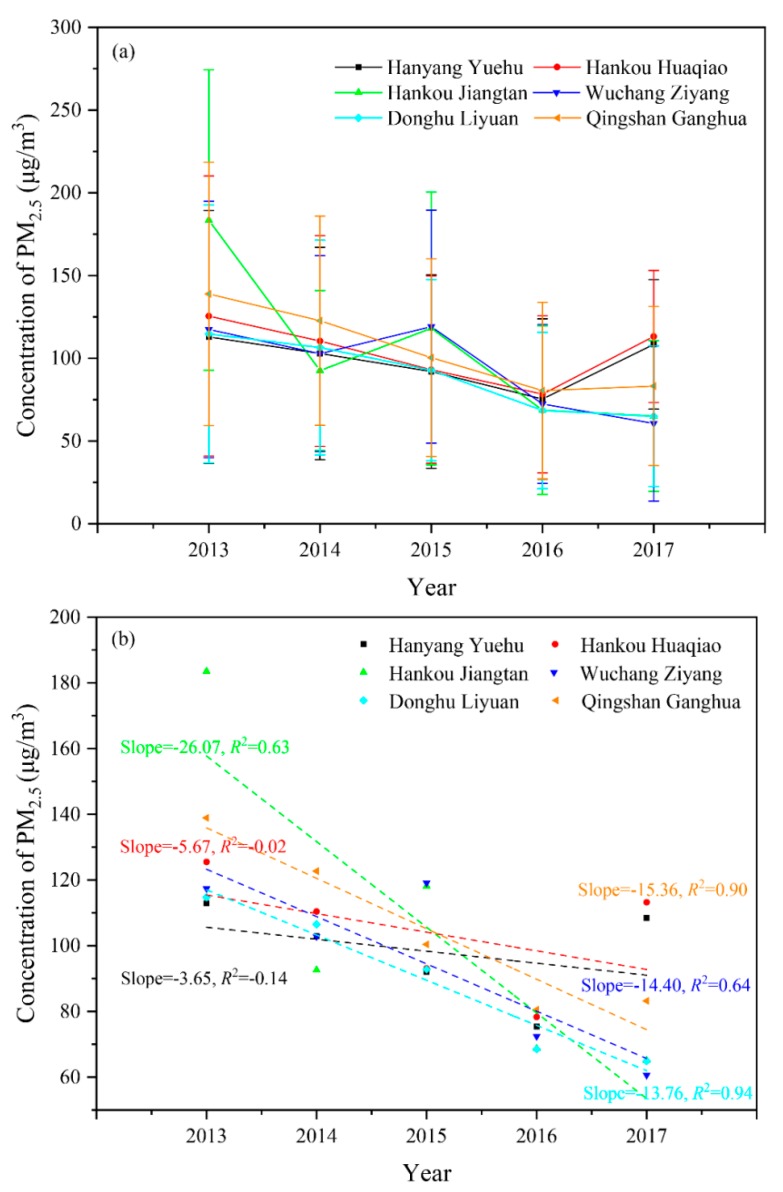
(**a**) Annual mean PM_2.5_ concentrations and standard deviations at various monitoring sites in the period of 2013–2017; (**b**) Linear trendlines at various monitoring sites in the period of 2013–2017.

Figure 2 shows the annual mean concentration, standard deviation, and linear trendlines for PM_2.5_ at various monitoring sites from 2013–2017. Using 5 years as the time scale, it can be observed that the PM_2.5_ concentration data from various monitoring sites showed fluctuations with an overall decreasing trend. The PM_2.5_ concentration data at the Hankou Jiangtan monitoring site showed the greatest fluctuation, with peaks and troughs alternating every year; the highest value of 183.5 μg/m^3^ in 2013 and the lowest value of 65.1 μg/m^3^ in 2017. For the Qingshan Ganghua monitoring site, the highest value of the PM_2.5_ concentration was 138.9 μg/m^3^ in 2013, and the lowest value was 80.5 μg/m^3^ in 2016. In the case of the Hankou Huaqiao monitoring site, the highest value of PM_2.5_ concentration was 125.5 μg/m^3^ in 2013, and the lowest value was 78.3 μg/m^3^ in 2016. However, a large increase in the PM_2.5_ concentration was observed in 2017; it reached a value of 113.2 μg/m^3^, and there was only a decrease of 12.3 μg/m^3^ in the five-year period. At the Hanyang Yuehu monitoring site, the highest value of PM_2.5_ concentration was 112.9 μg/m^3^, and the lowest value was 75.4 μg/m^3^. The Wuchang Ziyang monitoring site showed a decreasing value of PM_2.5_ concentration each year, with the highest value at 117.4 μg/m^3^. The Donghu Liyuan monitoring site showed decreasing PM_2.5_ concentrations each year, with the highest value at 114.7 μg/m^3^. The annual mean concentration reduction at the various monitoring sites were 23.68 μg/(m^3^·a), 11.14 μg/(m^3^·a), 2.46 μg/(m^3^·a), 0.90 μg/(m^3^·a), 11.36 μg/(m^3^·a), and 9.96 μg/(m^3^·a) for the Hankou Jiangtan, Qingshan Ganghua, Hankou Huaqiao, Hanyang Yuehu, Wuchang Ziyang, and Donghu Liyuan monitoring sites, respectively.

**Figure 3:**
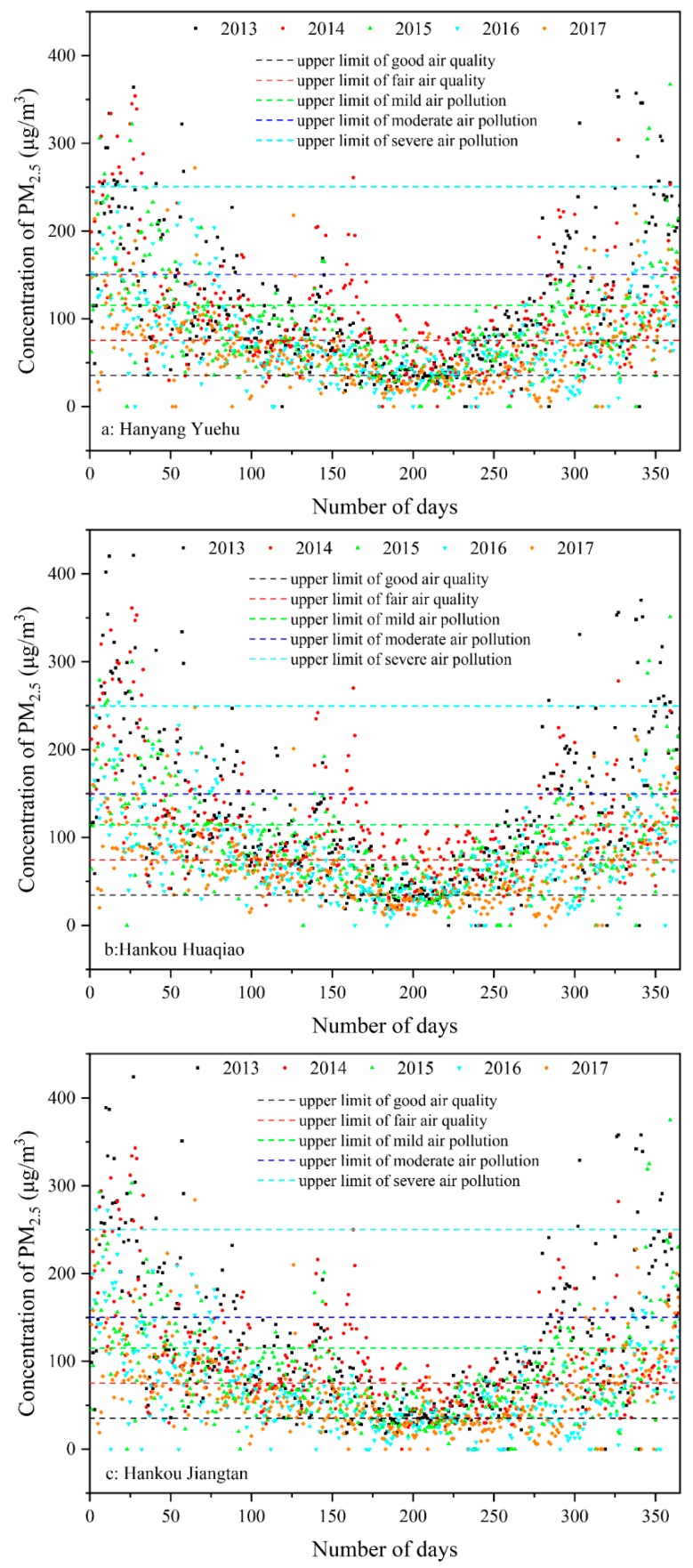

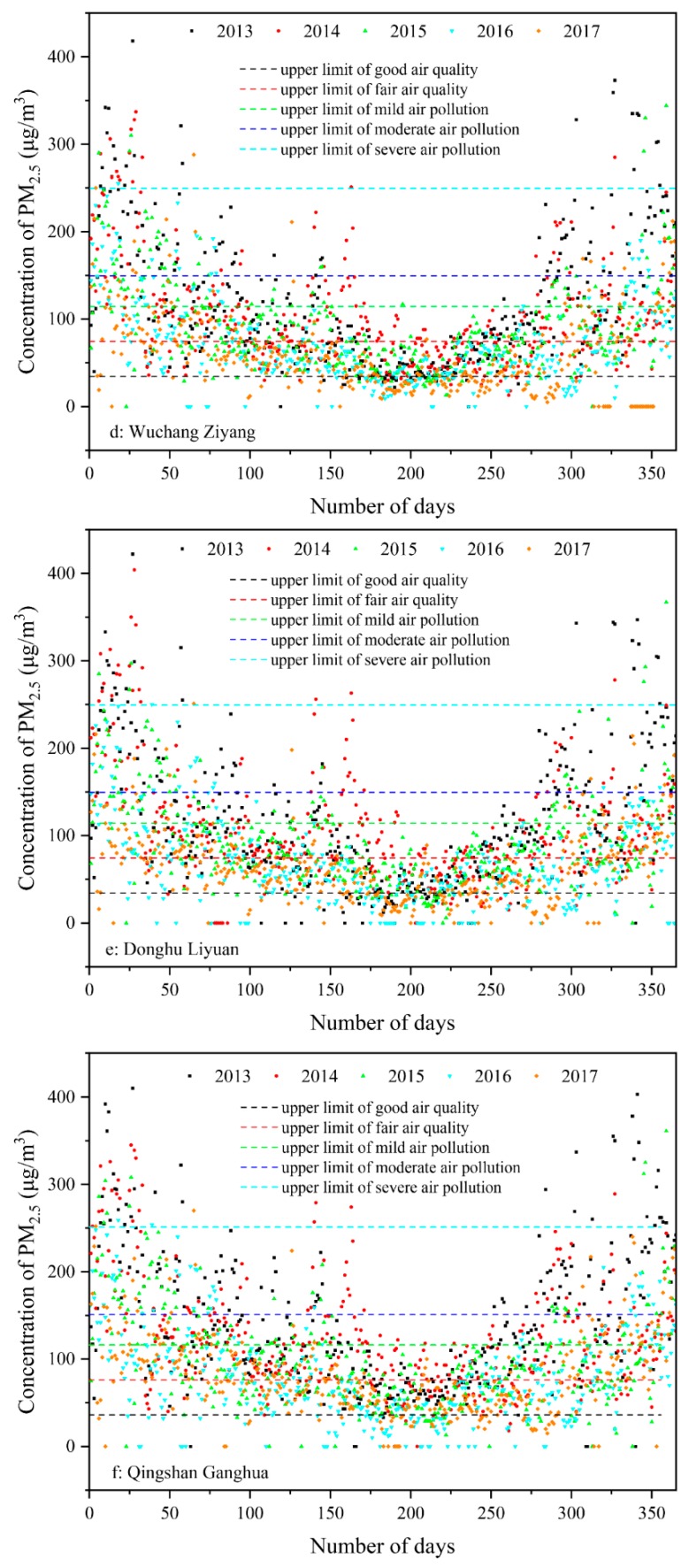
Annual variation trends of daily mean PM_2.5_ concentration at various monitoring sites during the period of 2013–2017.

Figure 3 shows the variation trend of the daily mean PM_2.5_ concentrations for various monitoring sites in 2013–2017. The PM_2.5_ concentration data from various monitoring sites in 2013–2017 were analyzed using 1 year as the time scale, in order to study the annual variation patterns in concentration. Overall, the daily mean PM_2.5_ concentration of every monitoring site shifts with a natural day and shows a saddle-like distribution.

From the overall variation trends, it can be observed that the PM_2.5_ concentration peaks showed a yearly declining trend, with the number of days of mild pollution gradually decreasing, while the number of days of severe and extreme pollution did not decrease, and the number of days with good air quality showed a slight increase. In the entire year, the PM_2.5_ concentration was between 35 μg/m^3^ and 150 μg/m^3^. This indicates that the PM_2.5_ concentration on most days was at a fair or moderate pollution level.

A natural day was used as the reference subject in order to analyze the PM_2.5_ concentration variation trends in the year. The “good air quality” points showed a scattered distribution in natural days throughout the year, were relatively concentrated at 175–300 days, and were less distributed at other natural day intervals. The “fair air quality” points showed a scattered distribution in natural days throughout the year, were relatively concentrated at 100–330 days, and were less distributed at 0–25 days and 325–365 days. The “mild air pollution” points showed a relatively uniform distribution in the natural day throughout the year, and were seldom at 175–225 days. The “moderate air pollution” points were concentrated at 0–175 days and 250–365 days, and seldom appeared in natural days, as can be seen in the middle of the graph. The “severe air pollution” points were concentrated at 0–80 days and 275–365 days. The PM_2.5_ concentration and degree of pollution showed clear patterns in natural day distribution throughout the year. This provides guidance for the governance of PM_2.5_ at various timings.

### 3.2. Monthly Variation Pattern of PM_2.5_ Concentrations

**Figure 4:**
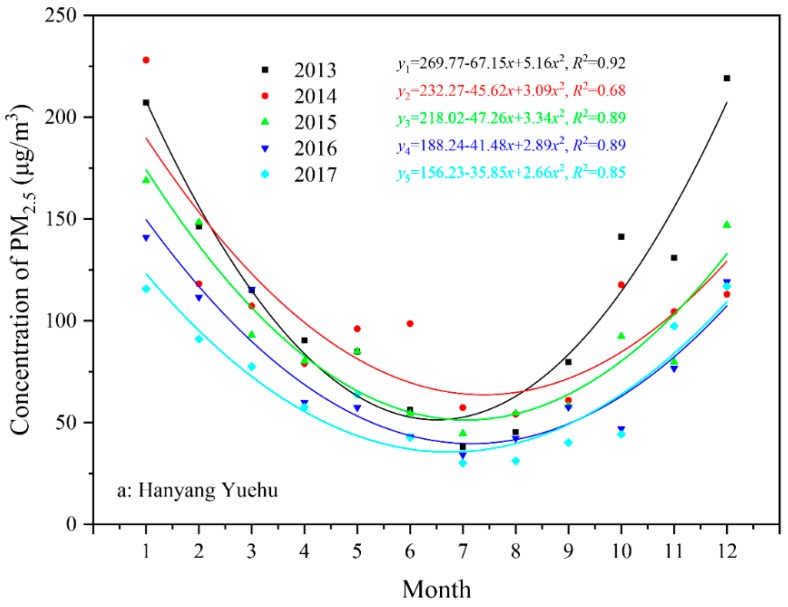

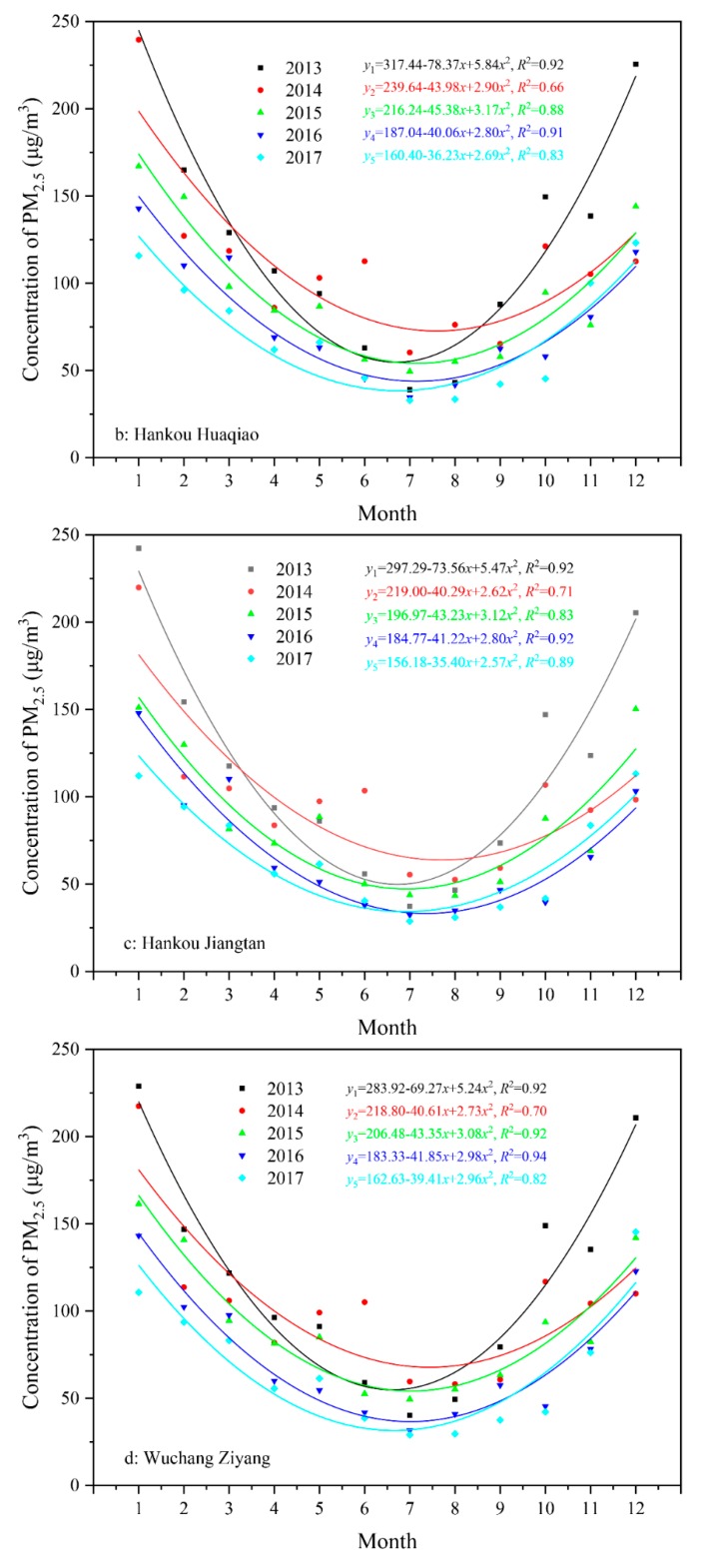

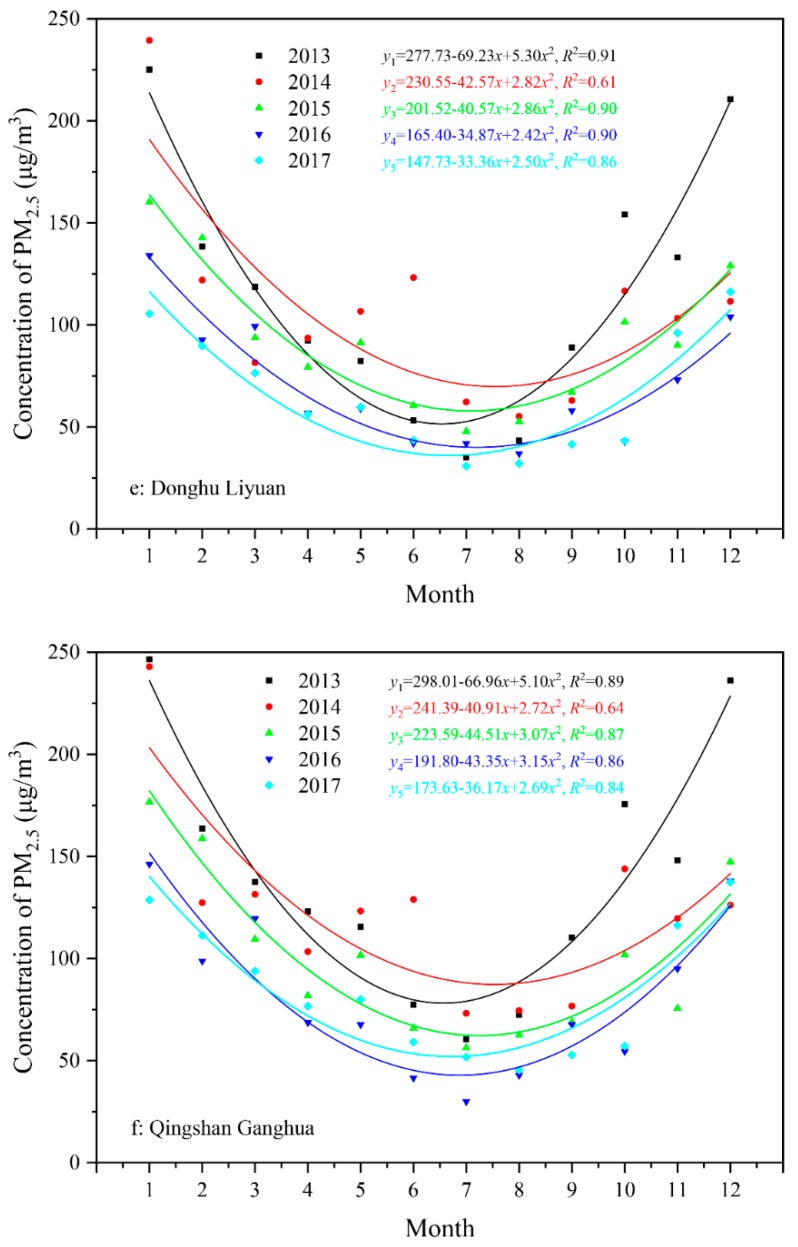
Variation trends of monthly mean PM_2.5_ concentration at various monitoring sites during the period of 2013–2017.

Figure 4 shows the variation trends of the mean monthly PM_2.5_ concentrations at various monitoring sites in 2013–2017. This result is consistent with the analysis results at Section 3.1. Using the mean monthly concentration as a reference standard, the PM_2.5_ concentrations showed a clear saddle-like distribution over the entire year. The binomial fitting standard formula *y* = *ax*^2^ + *bx* + *c* (where *y* is the dependent variable, *x* is the independent variable, and *a*, *b* and *c* are the constants determined by the specific data) was chosen to fit the data. Various fitting results showed that the mean monthly PM_2.5_ concentration showed a second-order function with an opening facing up. The symmetrical axis of the second-order function was concentrated in June, July, and August.

**Figure 5:**
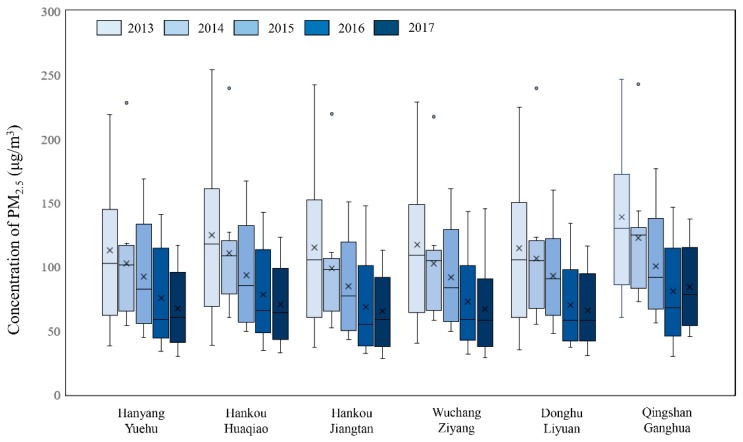
Highest and lowest values of monthly mean PM_2.5_ concentration at various monitoring sites during the period of 2013–2017.

Figure 5 shows the maximum and minimum values for the monthly mean PM_2.5_ concentrations at various monitoring sites in 2013–2017. In this five-year period, the highest PM_2.5_ concentration value for the Hanyang Yuehu monitoring site was 219.13 μg/m^3^ in December 2013, and the lowest value was 30.19 μg/m^3^ in July 2017. In the five-year period, the highest PM_2.5_ concentration value for the Hankou Huaqiao monitoring site was 254.26 μg/m^3^ in January 2013, and the lowest value was 32.87 μg/m^3^ in July 2017. The highest value for the Hanjou Jiangtan monitoring site was 242.23 μg/m^3^ in January 2013, and the lowest value was 28.74 μg/m^3^ in July 2017. The highest PM_2.5_ concentration value for the Wuchang Ziyang monitoring site was 228.87 μg/m^3^ in January 2013, and the lowest value was 29.06 μg/m^3^ in July 2017. The highest PM_2.5_ concentration value for the Donghu Liyuan monitoring site was 239.39 μg/m^3^ in January 2014, and the lowest value was 30.81 μg/m^3^ in July 2017. The highest PM_2.5_ concentration value for the Qingshan Ganghu monitoring site was 246.54 μg/m^3^ in January 2013, and the lowest value was 29.97 μg/m^3^ in July 2016. After 2013, the highest and lowest value for the monthly mean PM_2.5_ concentrations at the various monitoring sites for the entire year both showed an annual declining trend (with the exception of the lowest value in Qingshan Ganghua in 2017), and the highest and lowest values of the monthly mean PM_2.5_ concentrations in 2017 were reduced, as compared with those of 2013.

Table 3 shows the months in which the highest and lowest values of the monthly mean PM_2.5_ concentrations appeared from 2013 to 2017. It can be observed that the months with the highest monthly mean PM_2.5_ concentration were January (23 times) and December (7 times). The months with the lowest monthly mean PM_2.5_ concentration were July (23 times) and August (7 times). As compared with the other years, the month with the highest mean monthly PM_2.5_ concentration shifted from January to December. On combining the results of Figure 4 and Figure 5, it can be observed that January, October, and December are the three months with a relatively high monthly mean PM_2.5_ concentration in the entire year and the difference between the monthly mean PM_2.5_ concentration of October and the highest monthly mean concentration is generally less than 30 μg/m^3^. June, July, and August are the three months with a relatively low monthly mean PM_2.5_ concentration in the entire year, and the difference between the monthly mean PM_2.5_ concentration of June and the lowest monthly mean concentration is less than 15 μg/m^3^.

**Figure 6:**
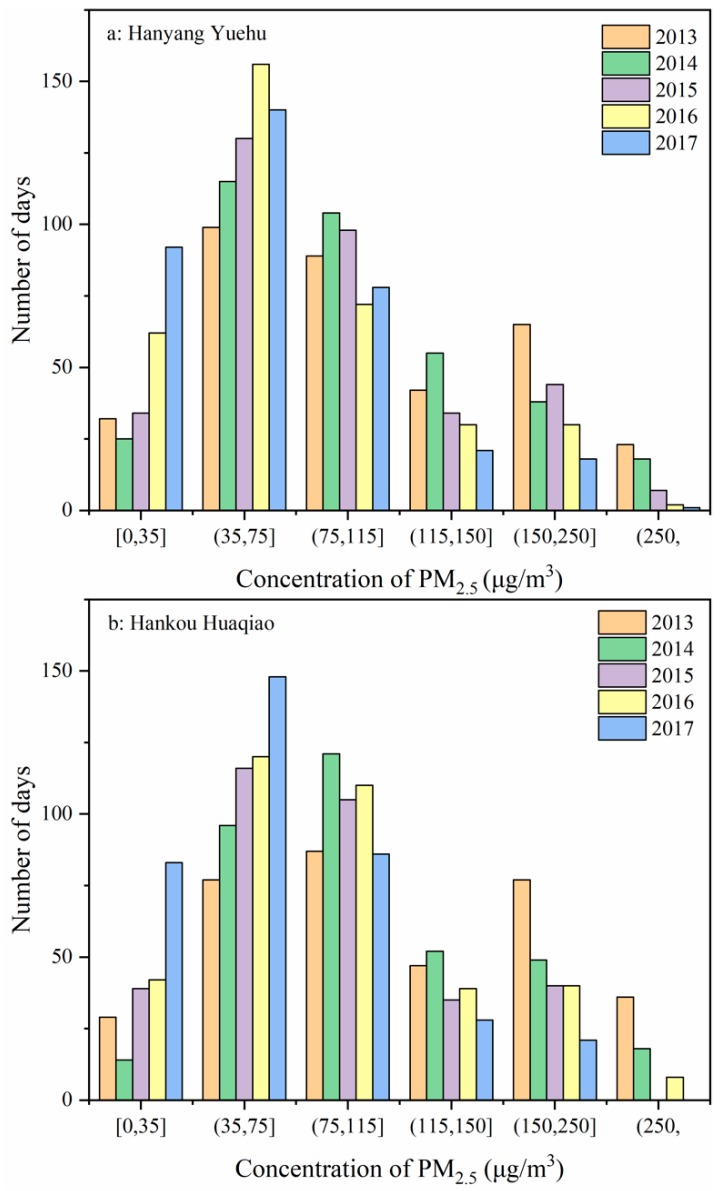

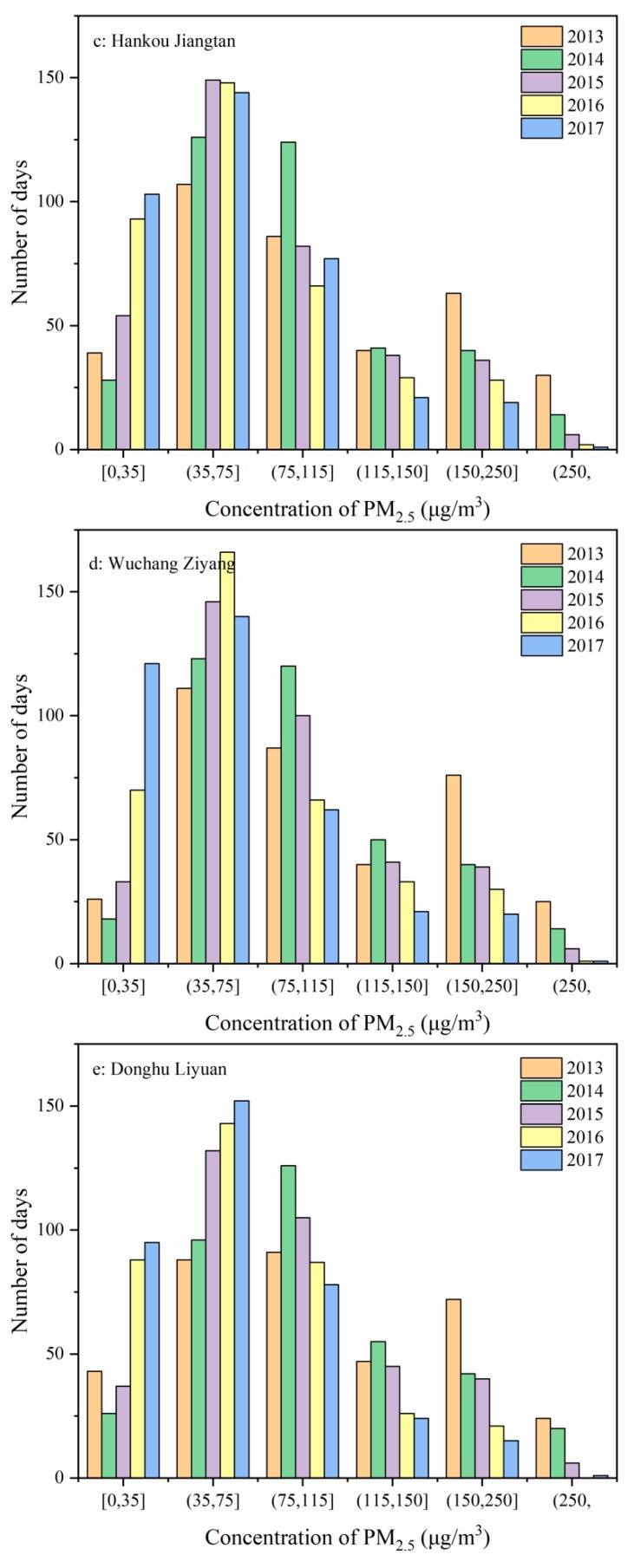

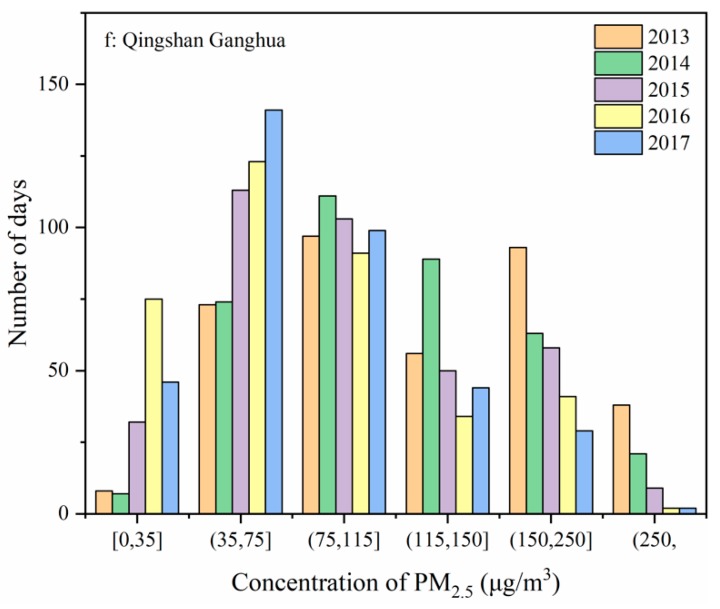
PM_2.5_ distribution at various monitoring sites in the period of 2013–2017.

Figure 6 shows the number of days with various PM_2.5_ evaluation intervals at the various monitoring sites in the five-year period. Overall, the number of days with the PM_2.5_ concentration interval of 35–75 μg/m^3^ was the highest, followed by the number of days with the concentration interval of 75–115 μg/m^3^. The number of days with the concentration interval of ≥250 μg/m^3^ was the least and the number of days with the concentration interval of 0–35 μg/m^3^ was also relatively low. At the same time, after 2013, the number of days with the concentration intervals of 115–150 μg/m^3^, 150–115 μg/m^3^, and ≥250 μg/m^3^ showed an annual decreasing trend. This data distribution shows that under the majority of circumstances, the daily mean PM_2.5_ concentration was at a “fair” (35–75 μg/m^3^) and “mild” air pollution (75–115 μg/m^3^) status in this region. In addition, the PM_2.5_ concentrations of these regions showed a decreasing trend. This is similar to the analysis results of the daily mean PM_2.5_ concentrations in Section 3.1. However, the over-limit status of the PM_2.5_ concentration was still serious as the number of days with a “good“ (0–35 μg/m^3^) and “fair” air quality (35–75 μg/m^3^) accounted for 40% of the entire year, and the overall PM_2.5_ concentration is still not ideal. Although this does not result in smog or affect traffic, long-term exposure to these PM_2.5_ concentrations can threaten human health, particularly that of children and the elderly.

### 3.3. Analysis of Contribution of PM_2.5_ to Air Pollution

**Figure 7:**
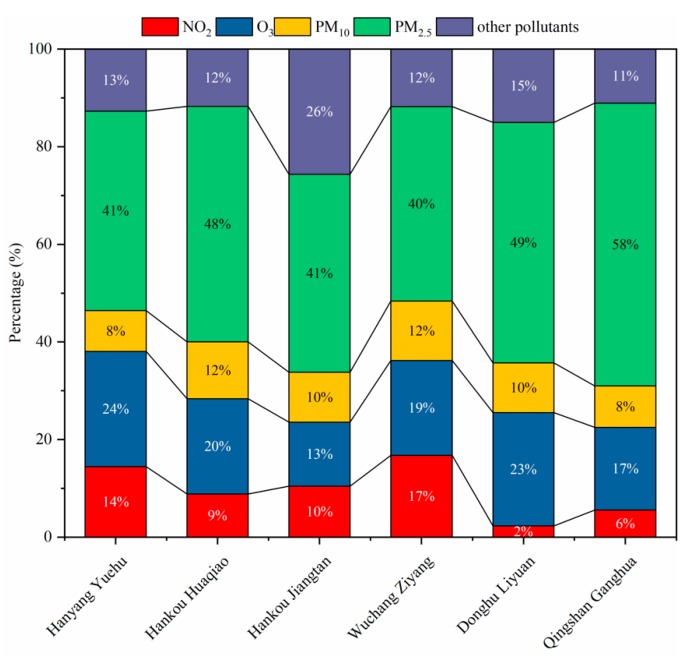
Percentage of major pollutants at various monitoring sites in the period of 2013–2017.

Figure 7 shows the percentage (a ratio of the number of days of a pollutant as the major pollutant to the number of days in the year) of major pollutants at various monitoring sites in 2013–2017. Overall, PM_2.5_ accounted for the greatest proportion of all the primary pollutants at all the stations, with an average proportion of more than 46%. The station with the lowest PM_2.5_ concentration value at 40% was the Wuchang Ziyang monitoring site, and the one having the highest value of at 58% was the Qingshan Ganghua monitoring site. This shows that PM_2.5_ is the primary air pollutant in Wuhan city, followed by O_3_ as the secondary pollutant with an average proportion of more than 19%. NO_2_ and PM_10_ have almost identical contributions to air pollution, with average proportions of 9.67% and 10%, respectively. The other pollutants are diverse and have a collective average proportion of 14.83%, which also contributes to the air pollution.

## 4. Conclusions

(1) In the period of 2013–2017, the PM_2.5_ concentration data from various monitoring sites showed fluctuations with an overall decreasing trend. The annual mean concentration reduction at various monitoring sites were 23.68 μg/(m^3^·a), 11.14 μg/(m^3^·a), 2.46 μg/(m^3^·a), 0.90 μg/(m^3^·a), 11.36 μg/(m^3^·a), and 9.96 μg/(m^3^·a) for the Hankou Jiangtan, Qingshan Ganghua, Hankou Huaqiao, Hanyang Yuehu, Wuchang Ziyang, and Donghu Liyuan monitoring sites, respectively.

(2) The various air quality points showed a scattered distribution on natural days throughout the year. The “good” air quality points were concentrated at 175–300 days and were less distributed in other natural day intervals. The “fair” air quality points were concentrated at 100—330 days and were less distributed at 0–25 days and 325–365 days. The “mild” air pollution points showed a relatively uniform distribution on natural days in the entire year and were few at 175–225 days. The “moderate” air pollution points were concentrated at 0–175 days and 250–365 days. The “severe” air pollution points were concentrated at 0–80 days and 275–365 days.

(3) The months with the highest monthly mean PM_2.5_ concentration were January and December. The months with the lowest monthly mean PM_2.5_ concentration were July and August (seven times). As compared with other years, the month with the highest mean monthly PM_2.5_ concentration in 2017 shifted from January to December. January, October, and December are the three months with a relatively high monthly mean PM_2.5_ concentration in the entire year, and the difference between the monthly mean PM_2.5_ concentration in October and the highest monthly mean concentration is generally less than 30 μg/m^3^. June, July, and August are the three months with a relatively low monthly mean PM_2.5_ concentration in the entire year, and the difference between the monthly mean PM_2.5_ concentration in June and the lowest monthly mean concentration is less than 15 μg/m^3^.

(4) The number of days with a daily mean PM_2.5_ concentration interval of 35–75 μg/m^3^ was the highest while the number of days with a daily mean PM_2.5_ concentration of ≥250 μg/m^3^ was the least. After 2013, the number of days with daily mean PM_2.5_ concentration intervals of 115–150 μg/m^3^, 150–115 μg/m^3^, and ≥250 μg/m^3^ showed an annual declining trend. However, the over-limit status of the PM_2.5_ concentration was still serious as the number of days with a “good” (0–35 μg/m^3^) and “fair” air quality (35–75 μg/m^3^) accounted for 40% of the entire year, and the overall PM_2.5_ concentration is still not ideal.

(5) PM_2.5_ accounted for a large proportion of major pollutants and is the main source of air pollution in Wuhan city with an average proportion of over 46%. The station with the lowest value of PM_2.5_ concentration of 40% was the Wuchang Ziyang monitoring site, the station with the highest value of 58% was the Qingshan Ganghua monitoring site, and the mean value of which was over 46%.

## Figures and Tables

**Table 1:** Relevant information of monitoring devices used.

Name of Device	Rating/Range	Precision	Specification
PM_10_ precollector	Da_50_ = 10 μm	±0.5 μm	Satisfied
PM_10_ sampling system	σ_g_ = 1.5 μm	±0.1 μm	Satisfied
PM_2.5_ precollector	Da_50_ = 2.5 μm	±0.2 μm	Satisfied
PM_2.5_ sampling system	σ_g_ = 1.5 μm	±0.1 μm	Satisfied
Medium flow meter	60–125 L/m	±2%	Satisfied

**Table 2:** Number of data points and validity rate for PM_2.5_ monitoring data at various stations.

**Year**	**Valid Data Hour (h)**					
	**Hanyang**	**Hankou**	**Hankou**	**Wuchang**	**Donghu**	**Qingshan**
	**Yuehu**	**Huaqiao**	**Jiangtan**	**Ziyang**	**Liyuan**	**Ganghua**
2013	8640	8640	8664	8688	8496	8592
2014	8664	8736	8688	8760	8544	8736
2015	8592	8568	8544	8688	8688	8592
2016	8592	8616	8208	8472	7992	8256
2017	8592	8688	8544	8544	8472	8472
**Year**	**Validity Rate of Hourly Data (%)**					
	**Hanyang**	**Hankou**	**Hankou**	**Wuchang**	**Donghu**	**Qingshan**
	**Yuehu**	**Huaqiao**	**Jiangtan**	**Ziyang**	**Liyuan**	**Ganghua**
2013	98.6	98.6	98.9	99.2	97	98.1
2014	98.9	99.7	99.2	100	97.5	99.7
2015	98.1	97.8	97.5	99.2	99.2	98.1
2016	97.8	98.1	93.4	96.4	91	94
2017	98.1	99.2	97.5	97.5	96.7	96.7

**Table 3:** Months with highest and lowest mean monthly PM_2.5_ concentration at various stations.

Monitoring Site	Month with Highest Mean Monthly PM_2.5_ Concentration					Month with Lowest Mean Monthly PM_2.5_ Concentration				
	2013	2014	2015	2016	2017	2013	2014	2015	2016	2017
Hanyang Yuehu	12	1	1	1	12	7	8	7	7	7
Hankou Huaqiao	1	1	1	1	12	7	7	7	7	7
Hankou Jiangtan	1	1	1	1	12	7	8	8	7	7
Wuchang Ziyang	1	1	1	1	12	7	8	7	7	7
Donghu Liyuan	1	1	1	1	12	7	8	7	8	7
Qingshan Ganghua	1	1	1	1	12	7	7	7	7	8
